# Impact of early antibiotic exposure on the risk of colonization with potential pathogens in very preterm infants: a retrospective cohort analysis

**DOI:** 10.1186/s13756-022-01110-1

**Published:** 2022-05-19

**Authors:** Caren Bubser, Jan Liese, Lina Maria Serna-Higuita, Andreas Müller, Matthias Vochem, Jörg Arand, Ulrich Karck, Maximilian Gross, Christian F. Poets, Christoph Härtel, Michael Zemlin, Christian Gille, Natascha Köstlin-Gille

**Affiliations:** 1 Department of Neonatology, Tübingen University Children’s Hospital, Calwerstrasse 7, 72076 Tübingen, Germany; 2 Institute of Medical Microbiology and Hygiene, Tübingen University Hospital, Tübingen, Germany; 3 Department of Clinical Epidemiology and Applied Biostatistics, Eberhard Karls University, Tübingen, Germany; 4 Department of Paediatrics, University of Bonn, Bonn, Germany; 5 Department of Neonatology, Klinikum Stuttgart Olgahospital/Frauenklinik, Stuttgart, Germany; 6 Clinic of Obstetrics and Gynecology, Klinikum Stuttgart Olgahospital/Frauenklinik, Stuttgart, Germany; 7 Department of Paediatrics, University of Würzburg, Würzburg, Germany; 8 Department of Paediatrics, Saar University Homburg, Homburg, Germany; 9 Department of Neonatology, Heidelberg University Children’s Hospital, Heidelberg, Germany

**Keywords:** MDRO, Early antibiotic exposure, Preterm infants, Dysbiosis

## Abstract

**Background:**

Sepsis is one of the most important complications in preterm infants. For this reason, most preterm infants receive antibiotics during their first postnatal week. Since 2013, a weekly colonization screening has been installed in German neonatal intensive care units (NICUs), including multi-drug resistant organisms (MDRO) and pathogens with increased epidemic potential. We here investigated the impact of early antibiotic exposure on the colonization with these pathogens.

**Methods:**

Data from 1407 preterm infants with gestational age < 32 + 0 weeks and born in three NICUs in Germany between January 2014 and December 2019 were analysed.

**Results:**

Antibiotics were administered to 911/1407 (64.7%) participating infants during their first postnatal week. Screening-targeted pathogens were detected in 547/1407 (38.9%). Early antibiotic exposure did not increase the risk of colonization with screening-targeted pathogens. The only independent risk factor for colonisation with potential pathogens was the admitting hospital. Interestingly, longer antibiotic therapy (> 7 days) decreased the risk for acquiring pathogens with increased epidemic potential.

**Conclusion:**

Early antibiotic exposure did not impact the risk for colonization with MDRO or highly epidemic pathogens in preterm infants. Further studies are needed to identify risk factors for the acquisition of MDRO and highly epidemic pathogens and potential associations with long-term outcome.

**Supplementary Information:**

The online version contains supplementary material available at 10.1186/s13756-022-01110-1.

## Introduction

Infections are a major cause of morbidity and mortality in preterm infants [1]. The latter have an increased vulnerability to infections because of an immaturity of their mucosal barriers and alterations in their immune responses compared to adults [2, 3]. For this reason, up to 80% of preterm infants receive antibiotics during their first postnatal week [4]. Although early antibiotics are essential in infants with proven infection, they may increase the susceptibility to adverse outcomes such as late onset sepsis (LOS), necrotizing enterocolitis (NEC), bronchopulmonary dysplasia (BPD), and death [4, 5], probably by altering the infants´ microbiota [6, 7].

Most neonatal infections involve mucosal surfaces. For both, neonatal sepsis and NEC, alterations in the intestinal microbiome were shown to precede disease onset, with causative pathogens often descending from the intestinal flora of infected infants [8–12]. This emphasizes the need for the prevention of gut dysbiosis in preterm infants.

As a consequence of several infection outbreaks in German neonatal intensive care units (NICUs), the German commission for hospital hygiene and infection prevention (KRINKO) issued updated guidelines for infection surveillance of very low birthweight (VLBW) infants in 2013, including weekly colonization screenings [13]. This weekly screening comprises (a) multi-drug resistant organisms (MDRO; in the following named class I pathogens), (b) bacteria with special pathogenicity (*Acinetobacter spp., Klebsiella pneumoniae, S. aureus*), which should be included in the screening when an invasive infection with one of these pathogens has been observed (in the following named class II pathogens) and (c) pathogens with increased epidemic potential (*Serratia marcescens, P. aeruginosa, Enterobacter spp.*) (in the following named class III pathogens).

The objectives of this study were to determine (1) if early empiric antibiotic therapy is a risk factor for the detection of screening targeted pathogens in preterm infants < 32 weeks gestation and (2) which other factors predispose to colonization with screening-targeted pathogens.

## Methods

The retrospective cohort analysis used prospectively collected data from all inborn infants born at < 32 weeks gestation and admitted to three NICUs (University Hospital Bonn, University Hospital Tübingen, and Olgahospital Stuttgart) in Germany from January 1, 2014 to December 31, 2019. It was approved by the ethics committees of each participating hospital (reference number 120/2019BO2 for Tübingen, B-F-2020–036 for Stuttgart and 161/21 for Bonn) including an informed consent waiver. The latter was granted because patients had been discharged home several years earlier, so that obtaining consent for study participation would have required a disproportionate effort. Demographic, clinical, and laboratory data were collected and entered into a database. The three participating units were chosen for pragmatic reasons (same documentation programs). No attention was paid to similar practices in the care of preterm infants when selecting the units.

Data on antibiotic therapy were collected for all infants from birth up to postnatal day 7. Any dose of an intravenously or orally administered antibiotic during this period was classified as “antibiotic therapy in the first week of life”. Empiric antibiotic therapy for early onset sepsis (EOS) consisted of ampicillin and tobramycin (unit 1), piperacillin and gentamicin (unit 2) and ampicillin + sulbactam and tobramycin (unit 3). Empiric antibiotic therapy for LOS consisted of ampicillin + amikacin + cefotaxime (unit 1), piperacillin + tazobactam and amikacin (unit 2), and piperacillin ± tazobactam alone or in combination with vancomycin or meropenem and vancomycin (unit 3). Other rarely used combinations, which deviated from the respective standard, were piperacillin ± tazobactam and tobramycin/gentamicin, mezlocillin and gentamicin.

Screening targeted pathogens (STP) were defined according to the recommendations of the German Commission for Hospital Hygiene and Infection Prevention (KRINKO). Class I pathogens (multi-drug resistant organisms) included Gram-negative rods with resistance to acylureidopenicillins and third/fourth generation cephalosporins (2MRGN), Gram-negative rods with resistance to acylureidopenicillins, third/fourth generation cephalosporins and fluoroquinolones (3MRGN), Gram-negative rods with resistance to acylureidopenicillins, third/fourth generation cephalosporins, fluoroquinolones and carbapenems (4MRGN) and methicillin-resistant *Staphylococcus aureus*. Class III pathogens included S*erratia marcescens, Pseudomonas aeruginosa,* and *Enterobacter spp.* Class II pathogens were not included in the analysis due to a large inter-hospital heterogeneity of the pathogens included in this group. From all infants included, weekly throat and anal/rectal swabs were taken and screened for the indicated STP [13]. Any pathogen detection up to and including day 35 of life was recorded. Since the aim of our study was to investigate the influence of early antibiotic therapy on the first phase of microbiome development, we chose the end of the first postnatal month and thus the end of the neonatal period as study endpoint. As routine swabs were taken only once a week, we included all positive swabs until day 35 to be sure that the end of the first postnatal month was recorded for all children. Children who died before the end of the observation period, i.e. before day 35, were excluded, as no statement about the colonisation up to day 35 was possible.

Data on maternal colonization with STP were collected whenever possible. Every maternal swab taken during a hospital stay in one of the participating hospitals within the last four weeks before birth was collected and compared to infants’ screening data.

### Definitions

#### Clinical parameters

*Colonization* with STP was defined as detection of a STP in at least one swab (anal/rectal or throat) until 35 days after birth. *Gestational age (GA)* was calculated from the best obstetric estimate based on early prenatal ultrasound and obstetric examination. *Small for gestational age (SGA)* was defined as birth weight < 10th percentile according to GA. *Antenatal exposure to antibiotic therapy* was defined as any antenatal antibiotic treatment of mothers up to seven days before birth. *Intraamniotic infection (IAI)* was defined according to the guideline of the German Society for Gynaecology and Obstetrics (DGGG) as maternal fever (≥ 38.0 °C), increased maternal inflammatory markers without any other cause (CRP > 10 mg/l or elevation of white blood cell count > 15,000/μL), foetal or maternal tachycardia, painful uterus and foul-smelling liquor. *Preterm premature rupture of membranes (pPROM)* was defined as rupture of membranes before 37 weeks of gestation. *Focal intestinal perforation (FIP)* was defined as the occurrence of spontaneous intestinal perforation with the need for laparotomy and a macroscopic diagnosis of isolated FIP by the attending surgeon.

#### Outcomes

*Neonatal sepsis* was defined as laid out by the national infection surveillance system “NEO-KISS” [14]; clinical sepsis was defined as sepsis with at least two clinical signs (temperature > 38 °C or < 36.5 °C, tachycardia > 200/min, new onset or increased frequency of bradycardias or apnoea, hyperglycaemia > 140 mg/dl, base excess <  − 10 mval/l, change in skin colour, increased oxygen requirements) and one laboratory sign (C-reactive protein > 10 mg/l, immature/neutrophil ratio > 0.2, white blood cell count < 5/nl, platelet count < 100/nl) and antibiotic treatment for ≥ 5 days, but no proof of causative agent in blood culture. Blood-culture confirmed sepsis was defined as clinical sepsis with proof of a causative agent in a blood culture [14]. Early-onset sepsis (EOS) and late-onset sepsis (LOS) were defined as blood-culture confirmed sepsis occurring within or following the first 72 h after birth.

*Retinopathy of prematurity (ROP) requiring surgery* was defined as stage 3–5 ROP requiring intervention (cryotherapy, laser therapy, or anti-VEGF treatment).

*Necrotizing enterocolitis (NEC) requiring surgery* was defined as clinical NEC classified as Bell Stage II or III with the need for laparotomy with or without resection of necrotic gut, and a macroscopic diagnosis of NEC.

*Bronchopulmonary dysplasia* (BPD) was defined as the need for oxygen supplementation or ventilation support at 36 weeks corrected age according to the NICHD criteria [15].

*Death* was defined as mortality during the primary hospital stay.

#### Statistical analysis

Data analyses were performed using SPSS 26.0 (IBM, Munich, Germany). Nominal and ordinal variables were described using relative and absolute frequency. Numerical variables were described as mean and standard deviation or median and interquartile range (IQR) according to the distribution of the data. Normality of the distribution was assessed by investigating skewness and kurtosis as well as QQ graphs, box plots, and histograms. Study populations were compared using univariate analysis. Continuous variables were evaluated using independent-samples t-test for normally distributed data or Mann Whitney test for non-normally distributed variables. Categorical variables were evaluated with Chi-square test. A *p* value of < 0.05 was considered statistically significant for single tests.

After univariate analyses, multivariate logistic regression models were used to identify independent risk factors for colonization with STP. Parameters with a *p* value ≤ 0.1 in univariate analysis were used as independent variables for the logistic regression model. Odds ratios (OR) and 95% confidence intervals (CI) were calculated. A subgroup analysis including only infants born < 28 weeks was performed. A *p* value of < 0.05 was considered statistically significant. Missing data were not imputed.

## Results

### Epidemiology of early antibiotic exposure

Of 1543 infants analyzed, 136 were excluded from the study as they died before day 35, so that a total of 1407 infants born at < 32 weeks’ gestation were included; 480 (34.1%) of these were born before 28 weeks. Table 1 shows the demographics of the study population. Of infants born before 32 weeks or 28 weeks, respectively 911/1407 (64.7%) and 428/480 (89.2%) were exposed to antibiotics within the first postnatal week. In the first postnatal week, culture proven sepsis was diagnosed in 67/1407 (4.8%) and 42/480 (8.7%) of infants born before 32 and 28 weeks, respectively.

**Table 1:** Demographics of the study population

	GA < 32 weeks (n = 1407, 100%)	GA < 28 weeks* (n = 480, 34.1%)
Gestational age (weeks)	28.8 ± 2.4	26.0 ± 1.4
Birth weight (g)	1150.3 ± 415.9	754.0 ± 209.8
Gender male (%)	720 (51.2)	237 (49.4)
Multiple pregnancies (%)	567 (40.3)	182 (37.9)
Admitting hospital		
I	627 (44.6)	222 (46.3)
II	314 (22.3)	91 (19.0)
III	466 (33.1)	167 (34.8)
Prenatal ABX (%)	510 (47.0; n = 1085)	221 (47.7; n = 463)
Antenatal steroids (%)	1245 (88.5; n = 1380)	424 (88.3; n = 473)
pPROM (%)	382 (31.7; n = 1204)	129 (31.3; n = 421)
Birth mode (%)		
Spontaneous	152 (10.8)	57 (11.9)
Elective C/S	711 (54.8)	241 (50.2)
Emergency C/S	482 (34.3)	182 (37.9)
APGAR 10	8.9 ± 1.0	8.6 ± 1.2
SGA (%)	214 (15.2; n = 1406)	90 (18.8; n = 479)
Mechanical ventilation (%)	474 (33.7)	303 (63.1)
Early ABX (%)	911 (64.7)	428 (89.2)
EOS (%)	38 (2.7)	24 (5.0)
FIP (%)	56 (4.0; n = 1403)	45 (9.4; n = 479)
NEC (%)	41 (2.9; n = 1406)	30 (6.3)
LOS (%)	139 (9.9)	98 (20.4)
BPD (%)	114 (8.1)	86 (17.9)
ROP (%)	228 (16.2; n = 1170)	177 (38.3; n = 462)
Death (%)	14 (1.0)	8 (1.7)

GA gestational age; pPROM premature preterm rupture of membranes; C/S caesarean section; SGA small for gestational age; ABX antibiotics; EOS early onset sepsis; FIP focal intestinal perforation; NEC necrotizing enterocolitis; LOS late onset sepsis; BPD bronchopulmonary dysplasia; ROP retinopathy of prematurity

*Subgroup analysis

### Detection of screening-targeted pathogens in very preterm infants

Screening targeted pathogens (class I or class III or both) were detected in 547/1407 (38.9%) study participants until day 35. Class I pathogens were detected in 215/1407 (15.3%) and class III pathogens in 488/1407 (34.7%). In infants born before 28 weeks, 182/480 (37.9%) were tested positive for targeted pathogens (class I or class III or both) until day 35 and 86/480 (17.9%) and 149/480 (31.0%) for class I and class III pathogens, respectively. Among infants who died before day 35 and were thus excluded, 9/136 (6.6%) were tested positive for any targeted pathogen (class I and/or class III), 6/136 (4.4%) for class I and 6/136 (4.4%) for class III pathogens. Half the detections of pathogens was observed until day 20. Additional file 1: Fig. S1A + B shows the time until first pathogen detection for class I and class III pathogens. Additional file 1: Fig. S2 shows pathogen distribution for class I pathogens. In class III pathogens, 87% were *Enterobacter spp.,* 9.5% *Serratia marcescens* and 3.5% *Pseudomonas aeruginosa.* The same pathogen was already detected in a prenatal maternal swab in 30/1082 (2.1%) of infants screened positive for targeted pathogens.

### Risk factors for colonization with potential pathogens

In the first week after birth, antibiotic exposure was not associated with risk of colonization with class I or class III pathogens in infants born before 32 or 28 weeks (Table 2).

**Table 2:** Detection of potential pathogens in infants with or without early antibiotic exposure

	GA < 32 weeks (n = 1407, 100%)			GA < 28 weeks (n = 480, 34.1%)*		
	No ABX (n = 496, 35.3%)	ABX (n = 911, 64.7%)	*p* value	No ABX (n = 52, 3.7%)	ABX (n = 428, 30.4%)	*p* value
At least 1 class I pathogen (%)	74 (14.9)	141 (15.5)	0.78	8 (15.4)	78 (18.2)	0.61
At least 1 class III pathogen (%)	168 (33.9)	280 (30.7)	0.23	17 (32.7)	132 (30.8)	0.79

GA gestational age; ABX antibiotics, *subgroup analysis

In infants born before 32 weeks, factors associated with the detection of class I pathogens in univariate analysis were gestational age, birth weight, admitting hospital, prenatal antibiotic exposure, pPROM, and mode of delivery (Additional file 2: Table S1). After adjustment for confounders, only admitting hospital remained an independent risk factor (Table 3). For class III pathogens, admitting hospital was the only independent risk factor (Additional file 2: Table S1 and Table 3).

**Table 3:** Multivariate analysis of factors associated with the detection of class I and class III pathogens

	GA < 32 (n = 1407, 100%)				GA < 28 (n = 480, 34.1%)*			
	Class I pathogens		Class III pathogens		Class I pathogens		Class III pathogens	
	(a)OR (95% CI)	*p* value	(a)OR (95% CI)	*p* value	(a)OR (95% CI)	*p* value	(a)OR (95% CI)	*p* value
Gestational age (weeks)	0.9 (0.8–1.0)	0.13	n.a	n.a	0.9 (0.7–1.1)	0.15	n.a	n.a
Birth weight (g)	1.0 (1.0–1.0)	0.61	n.a	n.a	n.a	n.a	n.a	n.a
Admitting hospital								
I	1		1		1			
II	1.4 (1.0–2.0)	0.08	2.1 (1.5–2.7)	**< 0.01**	1.8 (1.0–3.3)	0.05	n.a	n.a
III	0.1 (0.0–0.2)	** < 0.01**	1.5 (1.2–2.0)	**0.01**	0.1 (0.0–0.3)	**< 0.01**		
Prenatal ABX	1.2 (0.8–1.7)	0.47	n.a	n.a	1.3 (0.7–2.3)	0.53	n.a	n.a
pPROM	1.2 (0.8–1.9)	0.33	n.a	n.a	1.4 (0.8–2.5)	0.21	n.a	n.a
Mode of delivery								
Spontaneous	1				1		1	
Elective C/S	1.0 (0.5–1.8)	0.89	n.a	n.a	0.7 (0.3–1.7)	0.43	1.2 (0.6–2.3)	0.58
Emergency C/S	1.2 (0.7–2.1)	0.56			0.9 (0.4–1.9)	0.73	1.8 (0.9–3.5)	0.09

Significant *p*-values are printed in bold

GA gestational age; ABX antibiotics; pPROM premature preterm rupture of membranes; C/S caesarean section; FIP focal intestinal perforation. Corresponding umbers and *p* values from univariate analysis are shown in Additional file 2: Table S1. *subgroup analysis

In subgroup analysis of infants born before 28 weeks’ gestation, factors associated with detection of class I pathogens in univariate analysis were gestational age, admitting hospital, prenatal antibiotic exposure, pPROM, and mode of delivery (Additional file 2: Table S1). After adjustment for confounders, again only the admitting hospital remained an independent risk factor (Table 3). For class III pathogens, we found no dependent or independent risk factors (Additional file 2: Table S1).

### Impact of primary antibiotic therapy and duration of antibiotic exposure on colonization with potential pathogens

Lastly, we investigated the impact of primary antibiotic therapy (starting on the day of birth) and of the duration of early antibiotic exposure on the detection of STP. Antibiotic therapy was started on postnatal day 1 in 623/1407 (44.3%) of infants born before 32 weeks and 308/480 (64.2%) of those born before 28 weeks. We first performed univariate analyses with the same factors as listed in Additional file 2: Table S1, both for the start of antibiotic therapy on the first postnatal day and for the duration of antibiotic therapy (not shown). The variables that showed a *p* value of 0.1 or less in these analyses were then included in multivariate analysis, the results of which are shown in Table 4. In infants born at < 32 weeks’ and < 28 weeks’ gestation, no association between antibiotic exposure at the day of birth and detection of STP was found (Table 4). Duration of antibiotic therapy starting within the first week after birth had no significant effect on colonization with class I pathogens. However, prolonged antibiotic therapy (longer than seven days) reduced the risk of colonization with class III pathogens in infants born at < 32 weeks (Table 4).

**Table 4:** Impact of primary antibiotic therapy and duration of antibiotic exposure on detection of class I and class III pathogens in infants born < 32 weeks and < 28 weeks

	GA < 32 weeks (n = 911, 64.7%)				GA < 32 weeks (n = 911, 64.7%)			
	Adjusted for birthweight, prenatal ABX, pPROM, admitting hospital and mode of delivery				Adjusted for pPROM, admitting hospital and mode of delivery			
	No class I P (n = 770, 54.7%)	At least 1 class I P (n = 141, 10.0%)	OR (95% CI)	*p* value	No class III P (n = 631, 44.8%)	At least 1 class III P (n = 280, 19.9%)	OR (95% CI)	*p* value
ABX 1st day	525 (68.2)	98 (69.5)	1.0 (0.6–1.8)	0.89	425 (67.4)	198 (70.7)	1.1 (0.8–1.6)	0.58
ABX 1-2d (%)	250 (32.5)	46 (32.6)	1	0.36	194 (30.8)	102 (36.4)	1	0.09
ABX 3-7d (%)	411 (53.4)	79 (56.0)	1.4 (0.8–2.2)	0.22	344 (54.6)	146 (52.1)	0.8 (0.5–1.0)	0.13
ABX > 7d (%)	109 (14.2)	16 (11.3)	0.9 (0.4–2.1)	0.86	93 (14.7)	32 (11.4)	0.6 (0.3–1.0)	**0.04**

	GA < 28 weeks (n = 428, 30.4%)*				GA < 28 weeks (n = 428, 30.4%)*			
	Adjusted for GA, prenatal ABX, pPROM, admitting hospital and mode of delivery				Adjusted for pPROM and mode of Delivery			
	No class I P (n = 350, 24.9%)	At least 1 class I P (n = 78, 5.5%)	OR (95% CI)	*p* value	No class III P (n = 296, 21.0%)	At least 1 class III P (n = 132, 9.4%)	OR (95% CI)	*p* value
ABX 1st day	248 (71.0)	60 (76.9)	1.3 (0.6–2.8)	0.45	204 (68.9)	104 (78.8)	1.2 (0.7–2.2)	0.37
ABX 1-2d (%)	86 (24.6)	30 (38.5)	1	0.57	75 (25.3)	41 (31.1)	1	0.23
ABX 3-7d (%)	199 (57.0)	38 (48.7)	0.9 (0.5–1.7)	0.65	163 (55.3)	74 (56.1)	0.7 (0.4–1.2)	0.21
ABX > 7d (%)	65 (18.6)	10 (12.8)	0.6 (0.2–1.6)	0.29	58 (19.6)	17 (12.9)	0.6 (0.3–1.2)	0.11

Significant *p*-values are printed in bold

GA gestational age; ABX antibiotics. *subgroup analysis; adjusted for gestational age, birth weight, admitting hospital, prenatal ABX, pPROM and mode of delivery for class I pathogens and for admitting hospital, pPROM and mode of delivery for class III pathogens

## Discussion

In the present study, we investigated the impact of early exposure to antibiotics on the risk of colonization with potential pathogens in infants born before 32 weeks gestational age.

We found high rates of antibiotic treatment in the first postnatal week, with up to 90% of infants < 28 weeks exposed to antibiotics in our cohort. This is in line with data from Germany and the USA, reporting that more than 80% of VLBWI receive early antibiotics [4, 16]. Incidence of sepsis during the first week (EOS and LOS) was low in our cohort (4.8% for infants < 32 weeks) according to data from Germany [1] and other countries [17, 18]). In 28.1% of infants receiving antibiotics in the first postnatal week, therapy was terminated within 48 h, suggesting clinical resolution of symptoms and no increase in inflammation markers. This rate is much lower than that reported by Ehl et al., who showed that 53% of VLBWI with a clinical suspicion of infection had a negative CRP after 24–48 h [19], but similar to that observed by Stocker et al. (32% of infants with termination of antibiotic treatment within 48 h) [20]. These differences may be explained by the 20 year period elapsing between the two latter studies and ours, during which clinical practice has changed and more caution in prescribing antibiotics may have developed.

In our cohort, 39% of infants acquired at least one STP until day 35. Interestingly, the risk for colonization with STP did not increase with decreasing gestational age (38% for infants < 28 weeks). This finding was unexpected, since infants with lower gestational age have a more immature gastrointestinal and immune system and are at higher risk of invasive procedures, prolonged time until full enteral feeds and short-term complications like sepsis, FIP and NEC—factors that are associated with gut dysbiosis.

Within class I pathogens, *Enterobacter* spp. 2MRGN (27.9%) was the pathogen detected most often, followed by *Escherichia coli (E. coli)* 2MRGN (13.1%), and *Klebsiella spp.* 2MRGN (12.9%). Within class III pathogens, *Enterobacter* spp*.* predominated with 84%, which is in line with data from Korpela et al. showing that the preterm microbiome often is dominated by *Enterobacter* spp. [21]. Moreover, a recent single centre report from Germany showed that *E. coli*, *Enterobacter cloacae*, *Klebsiella oxytoca* and *Klebsiella pneumoniae* were the species most often detected in routine screening [22]. Although neonatal sepsis with *Enterobacter* spp*.* is comparatively rare [1, 23], it has been shown that a high relative abundance of *Enterobacter cloacae* is associated with NEC [24]. One limitation of our data is that we do not have information about swab location (anal/rectal or throat) for the screening results analyzed in this study so we cannot provide information on whether certain pathogens prefer certain colonization sites. This knowledge would be helpful to establish possible associations between colonization and the occurrence of certain diseases (e.g. colonization preferable in the throat and occurrence of BPD). Furthermore, we did not collect data on antibiotic resistance of pathogens involved in systemic infections in the infants of our cohort. Further studies are needed to investigate the impact of colonization with STP on short- and long-term neonatal outcome.

In 2018, a consensus group convened by the World Health Organization (WHO) created a priority list of non-mycobacterial antibiotic-resistant bacteria based on ten criteria (mortality, health-care burden, community burden, prevalence of resistance, 10-year trend of resistance, transmissibility, preventability in the community setting, preventability in the health-care setting, treatability, and pipeline). The scope of this work was to identify the most important resistant bacteria for which there is an urgent need for new treatments. Of the bacteria listed there, carbapenem-resistant gram-negative pathogens (4MRGN) (listed as priority 1: critical) were found in 10/1407 patients (0.7%) examined in our study. VRE and MRSA (listed as priority 2: high) were detected in 5/1407 (0.04%) and 9/1407 (0.6%), while other pathogens listed (*Helicobacter pylori, Campylobacter, Salmonella, Neisseria gonorrhoe, Haemophilus influenzae, Shigella*) were not included in the KRINKO-screening and could thus not be detected. The results show that, fortunately, infants in our cohort were very rarely colonized with pathogens against which therapeutic options are severely limited. Nevertheless, efforts should be made to prevent this in the future as well.

Contrary to our expectations, antibiotic exposure in the first postnatal week was not associated with colonization with STP until postnatal day 35. Even when the entire hospital stay was considered, there was no influence of early antibiotic therapy on the colonization with STP when the duration until detection of an STP was included as a confounder. Similarly, we found no effect of antibiotic therapy on STP colonisation when we considered all antibiotic administrations up to postnatal day 35 (data not shown). Several times, both in animal models [25] and human cohorts [26–28], it has been shown that early antibiotics cause prolonged perturbations to the intestinal microbiome and metabolome. Furthermore, early life exposure to antibiotics has been linked to adverse short- and long-term outcomes such as an increased risk of BPD or death [5], LOS [4], and NEC [29] as well as impaired growth [28], asthma development [30] and impaired immune responses upon vaccination [25]. Interestingly, we also found no impact of prenatal antibiotic exposure on colonization with STP. Here, it must be mentioned that the detection of an STP is not synonymous with the presence of dysbiosis. However, a "healthy" microbiome seems to prevent colonization by so-called pathobionts such as *Clostridium difficile* or VRE (reviewed in [31]). A study in adults also showed that a faecal microbiota transplantation supports the eradication of MRDO [32], so it seems reasonable to assume that the colonization of STP is also favoured by dysbiosis. However, further studies are needed to determine more precisely how the bacterial composition differs between children with and without STP, and whether the detection of STP actually indicates dysbiosis. An important limitation of our study potentially explaining the missing effect of early antibiotics on detection of STP in our cohort is that we did not consider antibiotic administration *after* the first postnatal week, probably also influencing microbiome composition. Furthermore, it is becoming increasingly clear that also drugs other than antibiotics have an impact on the human microbiome [33]. Further studies are needed to analyse the impact of single non-antibiotic therapeutics and their combinations on the microbiome of preterm infants.

De Man et al. showed that an antibiotic regime consisting of ampicillin and cefotaxime promoted development of antibiotic resistance in neonates when compared to a regime consisting of penicillin G and tobramycin [34]. Aminopenicillins had a selective effect towards resistant gram-negative bacilli. However, all participating units in our study had an aminopenicillin either in their EOS or in their LOS regime. De Man et al. also hypothesized that a combination of an aminopenicillin with cefotaxime may lead to an elimination of the normal intestinal flora by the aminopenicillin leading to an outgrow of gram-negative bacteria such as *Enterobacter* spp. and *Serratia* spp. that can degrade cefotaxime and aminopenicillins. One of the participating units used such a combination for LOS, which could possibly contribute to the observed center effect.

Concerning independent risk factors for detecting potential pathogens, we only identified the respective admitting hospital as a risk factor for colonization with STP. The fact that the bacterial spectrum is strongly dependent on the environment is obvious. Nevertheless, in various studies investigating risk factors for MDRO colonization in adult patients, the center factor was not analyzed at all [35, 36]. We observed that infants born spontaneously or via emergency cesarean section tended to have an increased risk of acquiring class III pathogens, while infants born via elective caesarean section more often acquired class I pathogens. While it is well known that infants delivered spontaneously have an altered microbiome compared to those delivered by caesarean section [37–39], we do not have any explanation for the observation that also the circumstances leading to caesarean section seem to impact microbiome composition. Since most emergency caesarean sections are accompanied by preterm labour and preterm labour in turn comes along with inflammation [40], it may be speculated that an inflammatory microenvironment favours colonization with *Serratia marcescens, P. aeruginosa,* and *Enterobacter spp.*

We found that in 2.1% of infants positively screened for targeted pathogens, the same pathogen had already been detected in prenatal maternal swabs. This number is comparatively low, but most likely underestimated as pathogens with resistance against acylureidopenicillins and third or fourth generation cephalosporins (2MRGN) were not marked as MRGN in maternal swabs and thus could not be included in analysis. The perinatal transmission of pathogens from the vaginal flora to the child is well known for group B streptococci [41], but has also has been shown for MDRO in about 27% of colonized mothers [42].

As another limitation of our study, it has to be mentioned that, unfortunately, we were unable to collect detailed nutritional data. Regarding the standard procedure in the three participating units, we can report that all three units started enteral feeding with colostrum or breast milk the day of birth. If there was no or too little breast milk available, two units (unit 1 and 2) used formula feeding and one unit (unit 3) used donor breast milk. Breast milk fortifiers were used by all units, with a cow's milk-based fortifier in units 1 and 2, and a human milk-based fortifier unit 3. These differences in the standard dietary regime could contribute to the observed centre effect in STP colonization. We were also unable to collect detailed data on the skin-to-skin contact of preterm infants in our cohort. Regarding this point, however, all participating units had the same standard procedure and started skin-to-skin as soon as infants seemed stable enough.

Data on the effect of the duration of antibiotic therapy on resistance are heterogenous. From prospective studies in older patients, there is evidence that a shorter duration of antibiotic therapy may reduce resistance rates [43, 44], but none of these studies investigated preterm infants. We found no effect of duration of antibiotic therapy started within the first postnatal week on resistance rates, but a reduced risk for colonization with *Enterobacter spp., Serratia marcescens* and *Pseudomonas aeruginosa*. This probably reflects the fact that most of the antibiotics used for empirical therapy in preterms are mainly effective in the gram-negative spectrum. Prospective studies investigating the impact of antibiotics on the microbiome, MDRO colonization and outcome of preterm infants are urgently needed.

## Conclusion

In conclusion, we provide data on the frequency of early antibiotic use and detection of MDRO and pathogens with high epidemic potential in preterm infants from a multicentre cohort of 1407 infants. We show that antibiotic treatment in the first postnatal week does not increase the risk of colonization with MDRO or highly epidemic pathogens in preterm infants. Interestingly, longer antibiotic treatment (> seven days) reduced the risk for colonization with highly epidemic pathogens until postnatal day 35. Our study contributes to a better understanding of colonization frequencies and kinetics in preterm infants and risk factors for colonization with potential pathogens. Further studies are needed to evaluate the impact of such colonization on neonatal outcome.

## Supplementary Information

**Additional file 1**. Figures.

**Additional file 2**. Additional tables.

## Data Availability

All data generated or analysed during this study are included in this published article and its supplementary information files. The datasets are available from the corresponding author on reasonable request.

## Abbreviations

ABX: Antibiotics
BPD: Bronchopulmonary dysplasia
EOS: Early-onset sepsis
FIP: Focal intestinal perforation
GA: Gestational age
IAI: Intraamniotic infection
KRINKO: German commission for hospital hygiene and infection prevention
LOS: Late-onset sepsis
MDRO: Multi drug resistant organism
MRGN: Multidrug-resistant gram-negative
NEC: Necrotizing enterocolitis
NICU: Neonatal intensive care unit
pPROM: Premature preterm rupture of the membranes
ROP: Retinopathy of prematurity
SGA: Small for gestational age
STP: Screening targeted pathogen
VLBW: Very low birthweight
VLBWI: Very low birthweight infant
