# Supplementary material for: Impact of early antibiotic exposure on the risk of colonization with potential pathogens in very preterm infants: a retrospective cohort analysis
Source: Antimicrob Resist Infect Control. 2022 May 19;11:72. doi: 10.1186/s13756-022-01110-1 (PMC9118610; doi:10.1186/s13756-022-01110-1)
Supplement: Supplementary file 1 — Additional file 1. Figures. [file 13756_2022_1110_MOESM1_ESM.pptx]

## Slide 1
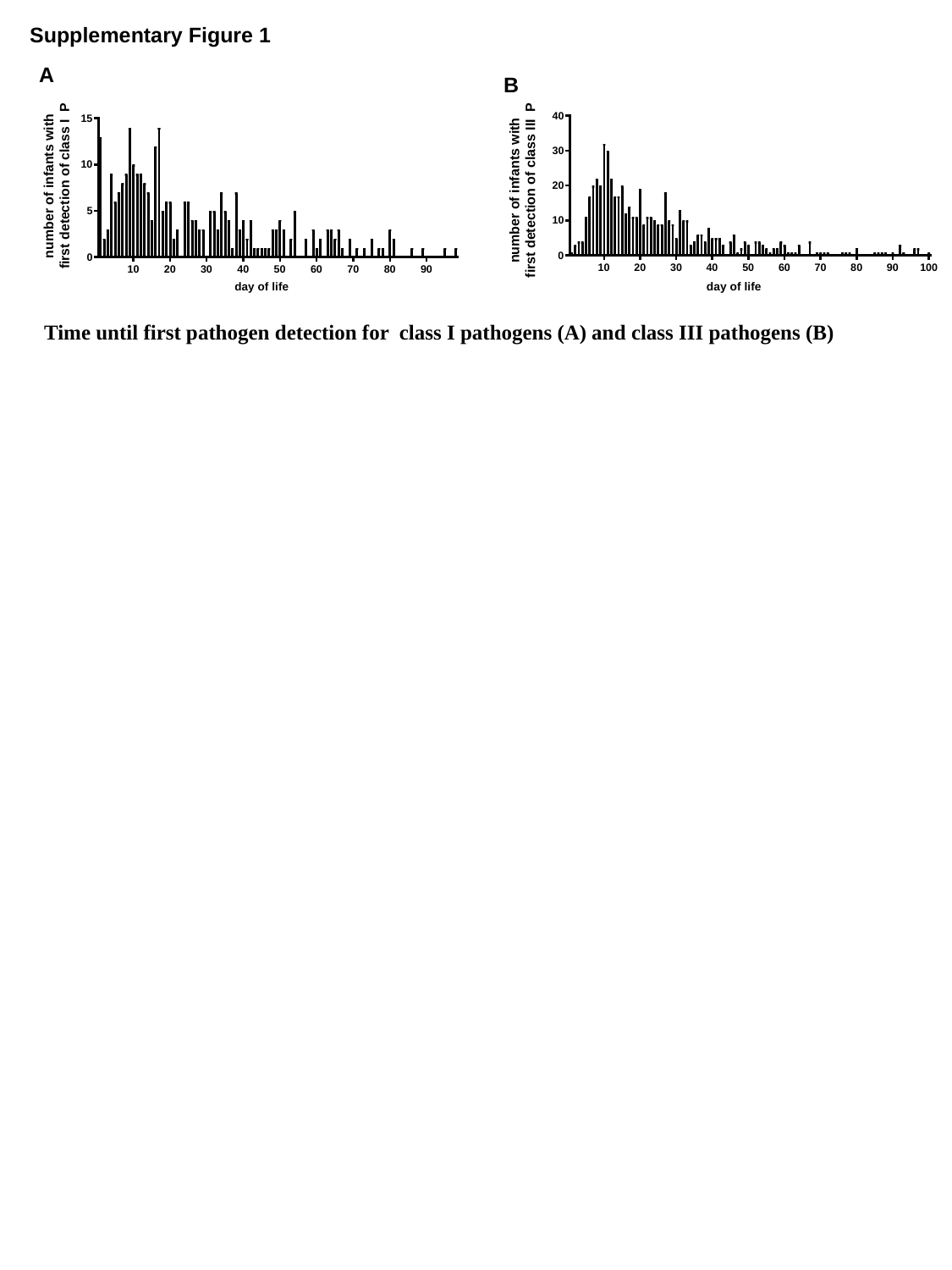

Supplementary Figure 1
A
B
Time until first pathogen detection for class I pathogens (A) and class III pathogens (B)

## Slide 2
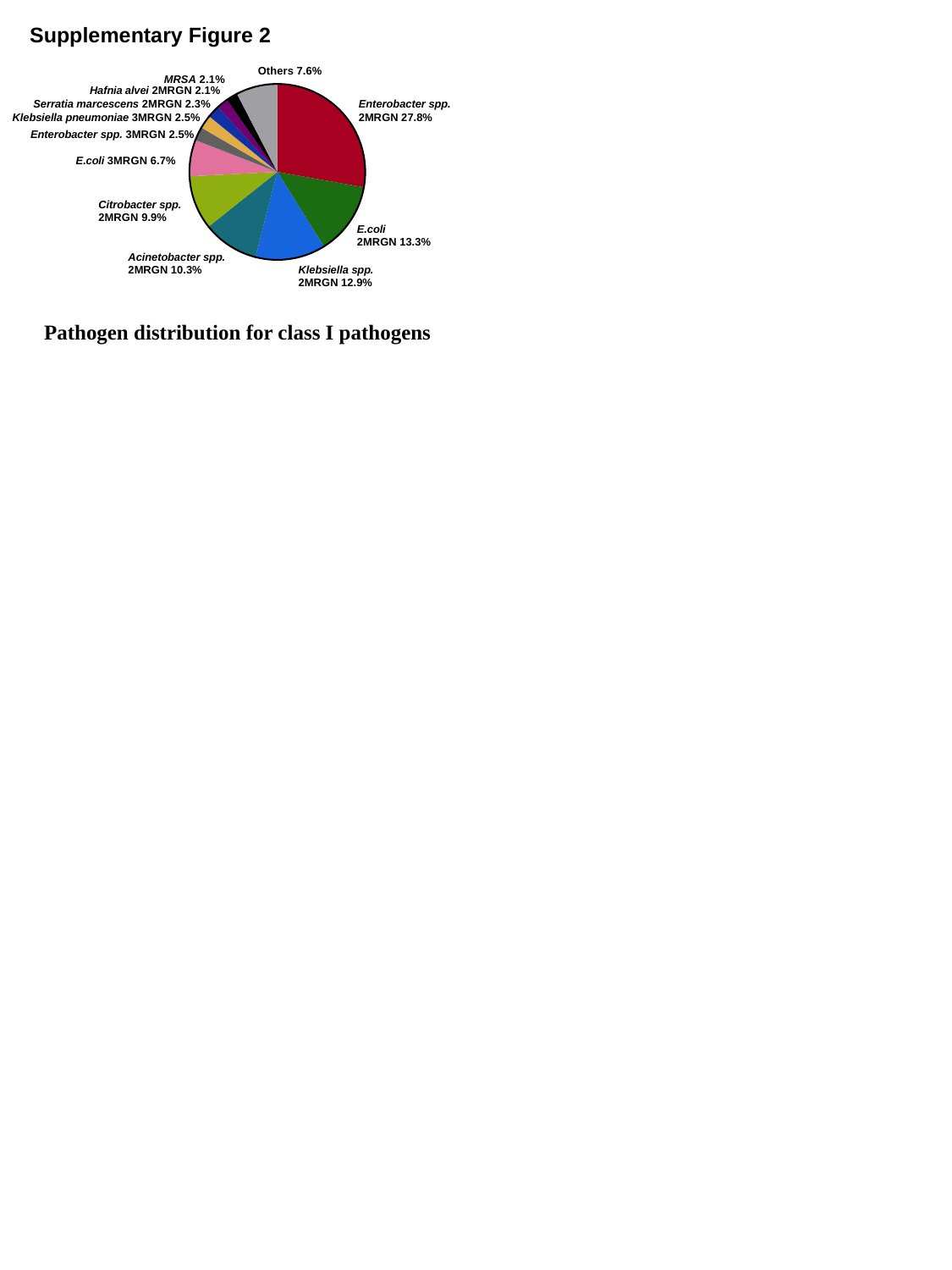

Supplementary Figure 2
Pathogen distribution for class I pathogens
