# Supplementary material for: Impact of early antibiotic exposure on the risk of colonization with potential pathogens in very preterm infants: a retrospective cohort analysis
Source: Antimicrob Resist Infect Control. 2022 May 19;11:72. doi: 10.1186/s13756-022-01110-1 (PMC9118610; doi:10.1186/s13756-022-01110-1)
Supplement: Supplementary file 2 — Additional file 2. Additional tables. [file 13756_2022_1110_MOESM2_ESM.docx]

**Supplementary Table 1: Characteristics of infants without and with the primary outcome of detection of class I and class III pathogens**

|  | GA <32 (n=1407) | | | GA < 28 (n=480) | | |
| --- | --- | --- | --- | --- | --- | --- |
|  | **No class I P (n=1192)** | **At least 1 class I P (n=215)** | **p-value** | **No class I P (n=394)** | **At least 1 class I P (n=86)** | **p-value** |

| Gestational age (weeks) | 28.9 ± 2.3 | 28.3 ± 2.6 | <0.01 | 26.0 ± 1.3 | 25.6 ± 1.4 | <0.01 |
| --- | --- | --- | --- | --- | --- | --- |
| Birth weight (g) | 1164.2 ± 421.2 | 1073.4 ± 377.0 | **<0.01** | 758.1 ± 206.6 | 735.2 ± 224.1 | 0.36 |
| Gender male (%) | 612 (51.3) | 108 (50.2) | 0.76 | 192 (48.7) | 45 (52.3) | 0.55 |
| Multiple pregnancies (%) | 486 (40.8) | 81 (37.7) | 0.39 | 155 (39.3) | 27 (31.4) | 0.17 |
| Center  I  II  III | 509 (42.7)  243 (20.4)  440 (36.9) | 118 (54.9)  71 (33.0)  26 (12.1) | **<0.01** | 170 (43.1)  59 (15.0)  165 (41.9) | 52 (60.5)  32 (37.2)  2 (2.3) | **<0.01** |
| Prenatal ABX (%) | 408 (45.5; n=897) | 102 (54.3, n=188) | **0.03** | 169 (44.7; n=378) | 52 (61.2; n=85) | **<0.01** |
| Early ABX (%) | 770 (64.6) | 141 (65.6) | 0.78 | 350 (88.8) | 78 (90.7) | 0.61 |
| pPROM (%) | 311 (30.5; n=1021) | 71 (38.8, n=183) | **0.03** | 98 (28.7; n=341) | 31 (43.7; n=71) | **0.01** |
| Mode of delivery  I  II  III | 123 (10.3)  677 (56.8)  390 (32.7)  (n=1190) | 29 (13.5)  94 (43.7)  92 (42,8) | **0.01** | 40 (10.2)  212 (53.8)  142 (36.0) | 17 (19.8)  29 (33.7)  40 (46.5) | **<0.01** |
| SGA (%) | 180 (15.1) | 34 (15.8) | 0.79 | 71 (18.1; n=393) | 19 (22.1) | 0.39 |
| FIP (%) | 51 (4.3; n=1189) | 5 (2.3) | 0.18 | 41 (10.4; n=393) | 4 (4.7) | 0.10 |
| EOS (%) | 31 (2.6) | 7 (3.3) | 0.59 | 19 (4.8) | 5 (5.8) | 0.78 |
|  | **No class III PP (n=959)** | **At least 1 class III PP (n=488)** | **p-value** | **No class III PP (n=331)** | **At least 1 class III PP (n=149)** | **p-value** |
| Gestational age (weeks) | 28.8 ± 2.4 | 28.8 ± 2.3 | 0.86 | 26.0 ± 1.4 | 26.0 ± 1.4 | 0.99 |
| Birth weight (g) | 1154.0 ± 419.6 | 1142.4 + 408.2 | 0.63 | 752.7 ± 204.0 | 756.8 ± 222.9 | 0.84 |
| Gender male (%) | 485 (50.6) | 235 (52.5) | 0.51 | 159 (48.0) | 78 (52.3) | 0.38 |
| Multiple pregnancies (%) | 390 (40.7) | 177 (39.5) | 0.68 | 128 (38.7) | 54 (36.2) | 0.61 |
| Center  I  II  III | 468 (48.8)  185 (19.3)  306 (31.9) | 159 (35.5)  129 (28.8)  160 (35.7) | **<0.01** | 155 (46.8)  57 (17.2)  119 (36.0) | 67 (45.0)  34 (22.8)  48 (32.2) | 0.34 |
| Prenatal ABX (%) | 358 (47.2; n=758) | 152 (46.5; n=327) | 0.82 | 148 (46.4; n=319) | 73 (50.7; n=144) | 0.39 |
| Early ABX (%) | 631 (65.8) | 280 (62.5) | 0.23 | 296 (89.4) | 132 (88.6) | 0.79 |
| PPROM (%) | 265 (32.2; n=824) | 117 (30.8; n=380) | 0.64 | 83 (29.1; n=285) | 46 (36.2; n=127) | 0.15 |
| Mode of delivery  I  II  III | 107 (11.2)  524 (54.6)  326 (34.0)  (n=957) | 45 (10.0)  247 (55.1)  156 (34.8) | 0.85 | 43 (13.0)  173 (52.3)  115 (34.7) | 14 (9.4)  68 (45.6)  67 (45.0) | 0.09 |
| SGA (%) | 144 (15.0; n=420) | 70 (15.6) | 0.77 | 59 (17.9; n=330) | 31 (20.8) | 0.45 |
| FIP (%) | 43 (4.5; n=955) | 13 (2.9) | 0.15 | 34 (10.3; n=330) | 11 (7.4) | 0.31 |
| EOS (%) | 27 (2.8) | 11 (2.5) | 0.70 | 19 (5.7) | 5 (3.4) | 0.27 |

GA gestational age; P pathogen; ABX antibiotics; pPROM premature preterm rupture of membranes; SGA small for gestational age; FIP focal intestinal perforation; EOS early onset sepsis
